# Vascular Disease in Systemic Lupus Erythematosus

**DOI:** 10.1155/2012/876456

**Published:** 2012-08-22

**Authors:** Athina Pyrpasopoulou, Sofia Chatzimichailidou, Spyros Aslanidis

**Affiliations:** 2nd Propedeutic Department of Internal Medicine, Hippokration General Hospital, Konstantinoupoleos 49, 54643 Thessaloniki, Greece

## Abstract

Vascular disease, either as a direct complication of the disease or developing as an accompanying comorbidity impairs significantly the quality of life of patients with SLE and represents the most frequent cause of death in established lupus. This paper aims to give an overview of the prevalence of the different forms of vasculopathy that can be encountered in a lupus patient, describe their pathogenesis, and address their impact on disease severity and outcome.

## 1. Introduction

Vascular disease is frequent in patients with systemic lupus erythematosus and represents the most frequent cause of death in established disease. In this context, vasculopathy can be directly aetiologically implicated in the pathogenesis of the disease, presenting as an acute/subacute manifestation of lupus (e.g., antiphospholipid syndrome, lupus vasculitis). Alternatively, it can develop as an important accompanying comorbidity (steroid-related atherosclerotic disease), or represent the synergistic pathogenetic outcome of augmented atherosclerosis within a proinflammatory environment.

This paper aims to give an overview of the prevalence of the different forms of vasculopathy that can be encountered in a lupus patient, describe their pathogenesis, and address their impact on disease severity and outcome.

## 2. Epidemiology

Systemic lupus erythematosus (SLE) is a multiorgan autoimmune disease with an increased incidence rate of thrombotic events (9–37%), occurring at a younger age compared to the control population [1].

The high rates of reported cardiovascular comorbidity [2] appear to be largely attributed to the significant predisposition of SLE patients for atherosclerosis and coronary artery disease (CAD) [3]; CAD is 5-6 times more frequent in SLE patients when compared to healthy controls, independent of other coexisting cardiovascular risk factors [4], and represents the majority of vascular events in SLE patients [5]. This excess risk is especially pronounced in women of younger age (35–44yrs; >50-fold) [6, 7].

Additionally, SLE patients are at a 20% higher risk for stroke; 3–15% experience a nonfatal thrombotic stroke [8], its incidence however usually recurrent and associated with a higher mortality rate than matched controls [9].

The rate of thrombotic events is higher in patients with disease of recent onset, when compared to patients with other autoimmune diseases and remains so throughout the course of the disease [10]; in the LUMINA study, which included multiethnic SLE patients of recent diagnosis, age, damage accrual at enrolment, and antiphospholipid antibodies, as well as the use of higher dosages of glucocorticoids were associated with a shorter time interval to thrombotic events [1]. Besides recorded clinical disease, subclinical involvement is significantly increased compared to healthy individuals, reaching 30% in reported studies [11].

Cardiovascular disease is the most frequent cause of death in established disease (>8 yrs after diagnosis; [12]): infection and renal involvement attributing for the majority of cases during the early stages (first year of diagnosis). As mortality rates of SLE patients tend to decrease significantly over time, and patients' life expectancy increases, its prevalence among an aged population becomes more pronounced, and its relevance gains increasing significance [13].

## 3. Atherosclerosis in Lupus

Atherosclerotic disease has been detected in lupus patients from cohort studies in a significant proportion of the population (≥30%), even when corrected for all other comorbidities [3, 14]. The prevalence was higher compared to corresponding groups, when patients included had previously been treated with atherogenically predisposing treatments, such as steroids, or surprisingly, in younger patients (age < 55 years).

Subclinical disease reached levels of 52% and was confirmed with a variety of techniques, such as carotid and brachial artery Doppler ultrasound, coronary artery angiography, and SPECT dual-isotope myocardial perfusion imaging [15].

Why do SLE patients develop more atherosclerosis? For once, cardiovascular risk factors seem to be more prevalent among SLE patients: hypertension, diabetes mellitus, sedentary lifestyle, hyperlipidemia, hypertriglyceridemia, hyperhomocysteinemia, and even premature menopause. However, as mentioned previously, after correcting for all predisposing cardiovascular risk factors, lupus still qualifies as an independent atherogenic (cardiovascular) risk factor [10, 16].

The SLE-associated proinflammatory cytokine burden and SLE-related treatments such as corticosteroids, that is, at a dose >10 mg/daily promote atherogenesis. These two parameters tend to interconnect, as disease of higher intensity and thus higher proinflammatory burden qualifies for the use of corticosteroids in lupus. Patients without previous history of steroid treatment tend to have more carotid atherosclerotic plaques, possibly due to less efficiently controlled disease activity. However, in lupus, aortic stiffness and increased pulse wave velocity do not always correlate with proven carotid atherosclerosis [17, 18]. As the pathogenesis of the disease unravels and more pro- and anti-inflammatory (i.e., protective) molecules become identified and their role in the course of the disease characterized, associations with increased aortic stiffness (e.g., C-reactive protein, VCAM, and C3) or vasoprotection (TGFb-1) become established [19].

## 4. Antiphospholipid Syndrome

The clinical antiphospholipid syndrome, an autoimmune syndrome usually developing in the context of systemic lupus erythematosus, is a condition defined as a predisposition for arterial and/or venous thromboses and/or recurrent miscarriages or other obstetric emergencies (premature birth, preeclampsia, etc.) in association with hematologic abnormalities and specific antibodies targeted against phospholipid-binding plasma proteins [20]. Indeed, retrospective studies in patients with clinical thrombotic events revealed a higher prevalence of positivity for antiphospholipid antibodies [21]. Thrombosis within the context of antiphospholipid syndrome may occur even in histologically normal vessels. However, in the majority of aPL-positive patients, seropositivity *per se* does not suffice for the development of clinical events. Thrombotic events seem to occur more readily in lupus patients with coexistent atherosclerosis [22]. Moreover, according to the second-hit hypothesis, a second trigger event—such as cigarette smoking, oral contraceptives, surgical procedures, prolonged immobilization Definition Immobilization refers to the process of holding a joint or bone in place with a splint, cast, or brace. This is done to prevent an injured area from moving while it heals, hypertension, or a genetic prothrombotic state increases the likelihood of an aPL-positive patient developing a vascular event [23–25].

Recently, the presence of microangiopathy, defined as capillary microhemorhages, and diagnosed with the aid of capillaroscopy, has been proposed as an augmentary screening tool for aPL-seropositive patients who are prone to develop clinical thrombotic manifestations [26].

## 5. Livedoid Vasculopathy: Thrombotic Disease and/or Vasculitis?

Besides overt vessel obstruction, vascular disease in lupus, especially when affecting medium- and small-sized vessels, may contain both vasculopathic and vasculitic pathophysiologic parameters.

Livedoid vasculopathy, a condition which can be observed in patients with systemic lupus erythematosus/antiphospholipid syndrome or specific forms of systemic vasculitis (mainly polyarteritis nodosa and cryoglobulinemia), is associated with chronic ulcerations of the lower extremities and characterized by uneven perfusion [27]. Initial isolated or focal petechiae or purpura progress to form asymmetrical, irregular, painful, and recurrent leg ulcers which heal in the form of atrophic white scars. In lupus in particular, analogous lesions may also be observed in the upper extremities (elbows) and seem to be associated with disease manifestations affecting other systems (e.g., central nervous system) [28].

The pathogenesis of livedoid vasculopathy has not been fully elucidated, or rather, cannot be solely attributed to a particular mechanism, as both hypercoagulable states, as well as autoimmune diseases, appear to associate with and contribute to its development [29].

The typical histological findings show dermal blood vessel occlusion [30] a diagnosis biochemically strengthened by increased prothrombotic markers (prothrombin gene variations, hyperhomocysteinemia, protein C deficiency, increased activity of plasminogen-activator-inhibitor, and activated protein C resistance). The histopathological findings of intravascular fibrin, segmental hyalinization, and endothelial proliferation clearly support the thrombotic parameter of its pathogenesis [31]. The presence of immunoreactants in the vessel wall and circulating immune complexes (such as rheumatoid factor) are in favor of its immunological component; the absence however of fibrinoid necrosis and inflammatory infiltration of the vessel wall differentiate livedoid vasculopathy from true vasculitides. The cutaneous features as well as nervous system manifestations (neuropathies or central nervous system involvement) in patients with livedoid vasculopathy may respond to immunosuppressive treatment.

## 6. Lupus Vasculitis

Distinction of inflammatory lupus vasculitis from antiphospholipid syndrome, which may present with similar clinical manifestations, is of major significance in terms of clinical management. Inflammatory vascular disease is triggered by the in situ formation, or the deposition, of immune complexes within the vessel wall.

Vasculitis may manifest in as high as 56% of lupus patients throughout their life, in contrast to antiphospholipid syndrome which has a prevalence of 15%. Patients with vasculitis are mainly male and tend to be of younger age [32].

Antibodies against endothelial cells have been identified as a major endothelial cell cytotoxic effector and have been implicated in the pathogenesis of several connective tissue diseases, predominantly vasculitides [33]. More than 80% of systemic lupus erythematosus patients are positive for antiendothelial cell antibodies (AECAs) [34, 35]. Antigens that react with AECAs include heparinlike compounds, DNA and DNA-histone complexes, ribosome proteins, elongation factor 1a, adenyl cyclase-associated protein, profilin II, plasminogen activator inhibitor, fibronectin, and b2-glycoprotein. AECA-endothelial cell interaction attracts monocyte adhesion and induces secretion of chemoattractant proteins and cytokines, thus triggering vasculitis.

Besides AECAs, the presence of antineutrophil cytoplasmic autoantibodies (ANCAs), mainly associated with primary systemic vasculitides, has also been reported in 15-20% of SLE patients [36].

Other forms of SLE-related vasculitis include drug-induced vasculitis [37] and infection-induced vasculitis [38] either through direct compromise of the vascular wall by pathogens, or through antigen-induced autoimmune and inflammatory processes.

## 7. SLE-Associated Vascular Disease: Burden and Outcome

In conclusion, vascular disease appears to be inherent in the pathogenesis and clinical manifestations of systemic lupus erythematosus; its severity ranging from mild cutaneous disease to severe organ dysfunction, such as renal or central nervous system vasculitis, atherosclerotic cardiovascular or cerebrovascular events, or catastrophic antiphospholipid syndrome.

In lupus, more readily than perhaps in any other systemic autoimmune disease, vascular disease may combine, in the same individual, atherosclerotic, and thrombotic disease with systemic vasculitis *per se*, and autoinflammatory-driven degeneration of the vessel wall.

In a histopathological study of autopsied SLE patients, histological characterization of vessel involvement and size of affected vessels were associated with the cause of death. Fibrinoid degeneration, intimal thickening, thrombosis, and sclerosis were identified and recorded. The principal manifestations of the disease were found to be associated with smaller-sized arteries. However, patients with medium-sized arterial involvement usually presented with more frequent thrombotic events and exhibited higher morbidity rates than the rest of the patients [39].

Lifestyle and pharmaceutical prevention measures, regular screening for subclinical disease, alertness for early clinical signs, accurate differential diagnosis, and targeted treatment may prove organ- or even life-saving especially in younger patients with more aggressive disease [40].
